# Anesthesiologic Management of Adult and Pediatric Patients with Obstructive Sleep Apnea

**DOI:** 10.3390/healthcare13172183

**Published:** 2025-09-01

**Authors:** Luigi La Via, Giannicola Iannella, Annalisa Pace, Giuseppe Magliulo, Giuseppe Cuttone, Rodolfo Modica, Mario Lentini, Carmelo Giancarlo Botto, Daniele Salvatore Paternò, Massimiliano Sorbello, Jerome R. Lechien, Antonino Maniaci

**Affiliations:** 1 Department of Anaesthesia and Intensive Care, University Hospital Policlinico “G. Rodolico-San Marco”, 95123 Catania, Italy; luigilavia7@gmail.com; 2 Department of Otolaryngology-Head Neck Surgery, La Sapienza University, 00161 Rome, Italy; annalisa.pace@uniroma1.it (A.P.); giuseppe.magliulo@uniroma1.it (G.M.); 3 Anesthesia and Intensive Care Unit, ‘Abele Ajello’ Hospital, ASP Trapani, 91026 Mazara del Vallo, Italy; giuseppe.cuttone@hotmail.it; 4 Asp 7 Ragusa, 97100 Ragus, Italy; rodolfo.modica@asp.rg.it (R.M.); mario.lentini@unikore.it (M.L.); giancarlo.botto@asp.rg.it (C.G.B.); paternomd@icloud.com (D.S.P.); massimiliano.sorbello@unikore.it (M.S.); antonino.maniaci@unikore.it (A.M.); 5 Department of Medicine and Surgery, University of Enna “KORE”, 94100 Enna, Italy; 6 Department of Surgery, UMONS Research Institute for Health Sciences and Technology, University of Mons, 7000 Mons, Belgium; jerome.lechien@umons.ac.be

**Keywords:** obstructive sleep apnea, anesthesia, perioperative management, CPAP, pediatric airway

## Abstract

Obstructive sleep apnea (OSA) is a prevalent yet underdiagnosed condition that significantly increases perioperative morbidity and mortality in both adult and pediatric populations. Its pathophysiology, involving intermittent upper airway obstruction during sleep, poses unique challenges for anesthesiologists due to altered airway anatomy, increased sensitivity to sedatives, and unpredictable ventilatory responses. This comprehensive review summarizes current evidence on the anesthesiologic management of OSA patients, focusing on preoperative screening, risk stratification, intraoperative considerations, and postoperative care. Effective management of OSA requires a multidisciplinary and individualized approach. Preoperative assessment should include validated tools such as STOP-Bang or polysomnography when available. Intraoperative strategies include careful titration of sedatives and opioids, airway protection techniques, and use of short-acting agents. Pediatric patients present specific anatomical and physiological risks, particularly in adenotonsillectomy cases. Postoperative monitoring, especially in the first 24 h, is critical to detect respiratory depression, with CPAP therapy often beneficial in selected patients. Recognizing and appropriately managing OSA in surgical candidates is crucial for improving outcomes and reducing complications. Anesthesiologists should tailor perioperative strategies to the severity of OSA, age group, and type of surgery. Future research should aim to refine predictive tools and establish standardized protocols, particularly in pediatric populations.

## 1. Introduction

Obstructive sleep apnea (OSA) represents a major perioperative risk factor, frequently underdiagnosed in adult and pediatric patients, and associated with a significant increase in postoperative morbidity and mortality [1]. Classically defined by recurrent episodes of upper airway collapse during sleep, OSA alters sleep architecture, induces intermittent hypoxemia, and causes autonomic and ventilatory instability, with cardiovascular, metabolic, and neurocognitive consequences [2]. In the surgical setting, the prevalence of OSA is notably higher compared to the general population, reaching up to 41% of high-risk elective surgical patients identified through STOP-BANG [3]. A critical element is that the majority (>80%) of patients with OSA are unaware of the disorder at the time of surgery [4]. Despite the evident clinical impact, the evolution of scientific evidence regarding perioperative anesthesiologic management has often been fragmented and heterogeneous between adults and pediatrics. Numerous studies have documented an increase in perioperative complications, including desaturation, difficult intubation, pneumonia, cardiac arrhythmias, and unexpected intensive care unit admissions [1,5]. In response, organizations such as the Society of Anesthesia and Sleep Medicine (SASM) and the American Society of Anesthesiologists (ASA) have developed guidelines on screening and preoperative management [6,7], while highlighting limitations related to the low quality of evidence on which they are based and the often-burdensome implementation in real hospital settings [6]. The pediatric literature, relatively more recent, has emphasized that adenotonsillectomy is the most frequent treatment for OSA in developmental age, but less than 10% of children undergo objective screening before surgery, with economical methods such as nocturnal oximetry preferred over complete polysomnography [8]. Moreover, in pediatric surgical patients, OSA status is not associated with significant differences in central respiratory depression following a single dose of opioid [9]. This context underscores a fundamental gap, as although guidelines exist their clinical application is often heterogeneous and not modulated to individual or pediatric risk profiles. SASM recommendations focus primarily on preoperative screening, while intra- and postoperative indications remain outdated, especially in pediatric patients [6]. Recent qualitative studies highlight that many anesthesiologic practices for children with OSA lack prospective evidence; for example, for components such as preoperative midazolam use, reduced fentanyl dose, and use of dexamethasone and non-opioid analgesia, there is variance among pediatric anesthesiologists and general current practice [10]. Another open issue concerns pediatric predictive tools; the recent CHASE-OSA model, developed on over 1300 children, proposes a combined score (craniofacial, adenotonsils, age, obesity, and symptoms such as frequent snoring and drowsiness) with an area under the ROC curve of 0.85 to identify moderate–severe OSA [11]. Although promising, there is uncertainty about universal applicability and its integration into preoperative clinical workflows as external validation remains limited [11]. Critically, many meta-analyses on perioperative complications in adult patients with OSA are based on retrospective studies or registries, limiting causal interpretability [5,6]. Recent studies highlight the lack of randomized studies on standardized perioperative management protocols, emphasizing methodological fragmentation between centers and populations [6]. These limitations also emerge in data consistency; for example, some studies on ambulatory patients suggest that OSA does not increase unexpected admissions in selected ambulatory procedures [1], while others highlight risks even in day-surgery contexts [5]. This dichotomy highlights the need for critical and contextualized evaluation of evidence, rather than generalized application of recommendations. For children, national guidelines (e.g., pediatric ASA) are weakly supported by randomized controlled trials, and most recommendations are based on observational case series and small-scale studies [10]. There is still no consensus on practical aspects such as the optimal location for postoperative monitoring, which saturation thresholds require ICU admission, or thresholds for CPAP use post-tonsillectomy [10]. In this context of divergences, fragmentations, and scarce robust evidence, this narrative review aims to offer a critical and integrated analysis of the main controversial areas in anesthesiologic management of patients with OSA, both adult and pediatric. The objective is to synthesize the hot topics in the literature, evaluate the quality and generalizability of evidence, and highlight the clinical and methodological gaps that hinder effective and personalized perioperative management in the light of recent diagnostic and predictive innovations [8,11,12]. Only with a critical approach to current recommendations and clinical practices can we evolve toward anesthesiologic strategies based on evidence and adaptive to different contexts and risk profiles.

## 2. Materials and Methods

### 2.1. Literature Search Strategy

The review was conducted in accordance with best practices for narrative syntheses of biomedical literature, with an emphasis on critical appraisal and integration of evidence relevant to adult and pediatric management of OSA patients. A comprehensive literature search was performed in PubMed/MEDLINE, Embase, Scopus, and the Cochrane Library from January 2000 to July 2025. Search terms included combinations of “pediatric”, “adult”, “sleeps apnea”, “OSA”, “anesthesia”, “management”, and “outcomes”, using both MeSH terms and free-text keywords.

### 2.2. Selection Criteria

We included peer-reviewed studies, systematic reviews, meta-analyses, clinical guidelines, and high-quality observational reports that addressed the anesthesiologic management of adult and pediatric OSA populations. Animal experiments, case reports, conference abstracts, and non-English publications were excluded. The retrieved studies were screened for methodological rigor, relevance, and applicability to anesthesia settings. Whenever available, we prioritized large-scale, multi-center, and high-quality prospective data. For controversial or evolving topics, contrasting findings were presented and critically analyzed to highlight strengths, limitations, and implications for clinical practice. Data was extracted independently by two reviewers and summarized narratively, given the heterogeneity in study designs, patient populations, and outcome measures. Quantitative data are reported descriptively, with emphasis on trends, magnitude of effects, and clinically meaningful endpoints.

## 3. Pathophysiology and Phenotypes of OSA Related to Anesthesia

A thorough understanding of OSA pathophysiology—for adult and pediatric populations—is essential to grasp the critical points of anesthesiologic management. This constitutes anatomic–physiological, phenotypic, and non-anatomical factors that influence perioperative risk, drug selection, and respiratory monitoring. Upper airway obstruction in OSA is caused by an altered balance between negative inspiratory forces and dilator musculature, particularly the genioglossus, which is unable to maintain oropharyngeal patency [13]. Anatomical burdens (obesity, tonsillar hypertrophy, retrognathia) make the airway more susceptible to collapse and increase the negative intrapharyngeal load [14,15]. Notably, recent studies highlight how genioglossus fatigue, both molecular and functional, constitutes a crucial mechanism in evolving OSA [16]. This aspect assumes clinical relevance in anesthesia as sedative agents and opioids further reduce muscle tone, accentuating the risk of dynamic obstruction during induction and emergence. However, this issue seems to be less relevant in pediatric patients, thus possibly causing harm when withholding opioids [9]. Recent evidence also highlights that the consequences of OSA extend beyond respiratory mechanics, with systemic implications that intersect with perioperative risk. Intermittent hypoxemia and sleep fragmentation contribute to sustained oxidative stress and low-grade inflammation, which in turn have been linked not only to cardiovascular and metabolic sequelae but also to neurocognitive dysfunction and sensory impairment, including hearing loss [17,18,19]. These findings suggest that anesthesiologists should consider OSA as a multisystem disorder; inflammatory burden may increase susceptibility to perioperative complications such as impaired wound healing and exaggerated stress responses, while neurocognitive vulnerabilities could amplify postoperative delirium or delayed recovery. In pediatric patients, the interplay between adenotonsillar-related airway obstruction, recurrent hypoxemia, and neurocognitive consequences reinforces the need for tailored perioperative monitoring strategies that extend beyond respiratory parameters alone. In summary, patients with OSA can be distinguished into various pathophysiological phenotypes, based on anatomical collapsibility, CO_2_ sensitivity (which involves reduced ventilatory reactivity), arousal threshold (which moderates response to respiratory events), neuromuscular function, and airway wall compliance. A recent phenotyping model describes these traits as predictors of both clinical severity and response to therapies such as CPAP or MAD (mandibular advancement device) [20]. The importance of this approach emerges in anesthesiology, where the phenotype conditions the response to sedation, mechanical ventilation, hypoxic stress propagation, and airway pliability. In pediatric patients, OSA pathogenesis is equally complex but with distinct features; adenotonsillar disease is frequently the primary cause, sometimes associated with obesity and craniofacial anomalies. Standard models describe greater mechanical airway collapsibility, greater ventilatory reactivity, and often a lower arousal threshold compared to adults [10]. A recent update examined the neurophysiology of SDB in children, highlighting how intermittent hypoxemia and sleep fragmentation lead to neurocognitive and behavioral consequences that also have anesthesiologic implications in the perioperative period (increased risk of laryngospasm, bronchospasm, and respiratory depression) [1]. This creates a critical risk terrain where the anesthesiologist must consider differentiated phenotypes to dose medications and predict respiratory events. Thus, the main contrast between adults and children lies not only in anatomical mechanisms but also in ventilatory regulation and response to anesthesiologic intervention. Adults with high CO_2_ sensitivity and high arousal thresholds might better tolerate light inductions but risk prolonged periods of apnea if opioids or sedatives depress their ventilation [14]. Conversely, in children with low arousal thresholds and reduced muscular ventilatory reserves, even moderate doses of sedatives can trigger apnea and early desaturation [1]. Common errors in the literature include applying adult-based protocols to pediatric patients without phenotypic adaptation, generating ambiguous results in retrospective analyses. Critically, many therapeutic approaches—especially CPAP and oral devices—do not adequately differentiate these phenotypes and are often validated only in selected patient subgroups. A recent head-on analysis requires a paradigm shift as therapy must be personalized based on phenotype, as it must also be in the anesthesiologic setting, choosing different strategies for patients with predominant anatomical collapsibility compared to those with predominance of ventilatory instability or altered arousal threshold [20]. This model opens new scenarios for research as well; laboratory tests and PSG algorithms integrated with machine learning models could early identify phenotypes and allow modulation of induction, sedation, ventilation, and analgesia based on individual respiratory profiles [4]. In the clinical setting, however, data remain limited. The most recent pediatric reviews note that robust prospective studies are lacking on the relationship between pathophysiological phenotype and anesthesiologic outcome: most derive from clinical case series and hospital registries with sudden exposure to drugs and non-standardized monitoring [3,10]. Furthermore, clear definition of phenotypic parameters (e.g., arousal threshold, CO_2_ reactivity, measurable PSG collapsibility) is not uniformly reported in clinical protocols. This makes it difficult to extrapolate universal recommendations, reducing the effectiveness of strategies centered only on BMI or AHI.

## 4. The Challenge of Preoperative Evaluation

One of the main difficulties in perioperative management of patients with OSA is represented by underdiagnosis, which persists despite established screening tools (Table 1).

Recent studies highlight that anesthesiologists often do not perform systematic evaluations, especially in pediatric ambulatory settings: in a double-blind study, only a minority of practitioners applied screening tools before pediatric ambulatory surgical procedures, highlighting a gap between recommendations and practice [24]. In the adult field, the STOP-Bang questionnaire is the most commonly used. Thanks to its high sensitivity—up to 95% for moderate–severe OSA with scores ≥ 3—it is considered effective for “ruling out” high-risk patients [7,25]. However, the high sensitivity is accompanied by low specificity, which can generate an excess of false positives, with consequent arbitrary prolongation of the preoperative workup or unnecessary diagnostic investigations [22]. A cutoff ≥ 4 improves specificity in obese or bariatric populations, without compromising sensitivity too much [21]. But the definition of the optimal score for perioperative clinical use remains controversial [21,26]. Emerging critical issues also concern the utility of the Berlin Questionnaire or P-SAP; few studies propose them in anesthesiologic contexts, and their direct comparison with STOP-Bang does not show decisive superiority [23]. Some authors suggest two-phase strategies, in which a STOP score ≥ 3 followed by additional clinical evaluations (e.g., BMI, neck circumference, serum bicarbonates) allows for more precise risk stratification [25]. For pediatric patients, the situation is even more problematic: there are no validated screening tools for large-scale preoperative use. This hinders accuracy and timeliness in perioperative management. In pediatric dental contexts, for example, there is a complete lack of a dedicated screening tool to recognize at-risk patients before sedation or intervention [27]. Even in hospitals, routine use of questionnaires is sporadic, and recourse to polysomnography—the diagnostic gold standard—is limited to a few selected cases, often for practical or organizational reasons [28]. An additional critical element concerns the external validity of screening tests in contexts different from those in which they were designed: many models and scores derive from studies on adult Western populations in university centers with high BMIs. Their performance in pediatric patients or adults with heterogeneous clinical profiles is poorly documented [29]. This raises questions about possible selection bias and generalizability. A recent study has nonetheless attempted to validate innovative tools, for example, smartphone-based devices or deep learning applications—using microphones and accelerometers to estimate AHI—have shown high sensitivities (>0.9) in adults with AHI > 15 or >30 [26]. Similar emerging approaches are very promising but still need to address problems of standardization and clinical implementation in preoperative environments [30]. From an operational standpoint, the literature reveals divergences between SASM/ASA guidelines and real-world studies, as guidelines recommend systematic screening but clinical validity in terms of reducing perioperative complications is not completely proven. Some works report that screening does not directly reduce adverse outcomes, but allows targeted interventions (e.g., preoperative CPAP or prolonged hospitalization) that could mitigate risks [26]. However, randomized trials on preoperative populations are scarce, limiting the strength of recommendations [21]. Another controversial point is the interval between screening and surgical intervention. In patients with high scores, referral for polysomnography can lead to unjustified delays or an excessive number of diagnostic studies on patients already known to be at high clinical risk. Part of the literature suggests rapid procedures (e.g., home-based or nocturnal oximetry), but comparative validity remains low [28,29]. Furthermore, questions emerge about the appropriateness of STOP-Bang cutoffs in obese populations or those with hypoventilation. Serum HCO_3_^−^ values ≥ 28 mmol/L, for example, have been proposed as additional markers to identify severe OSA [25], but their practical integration is questionable in resource-limited contexts.

## 5. Risk Stratification and ASA Guidelines

**Figure 1:**
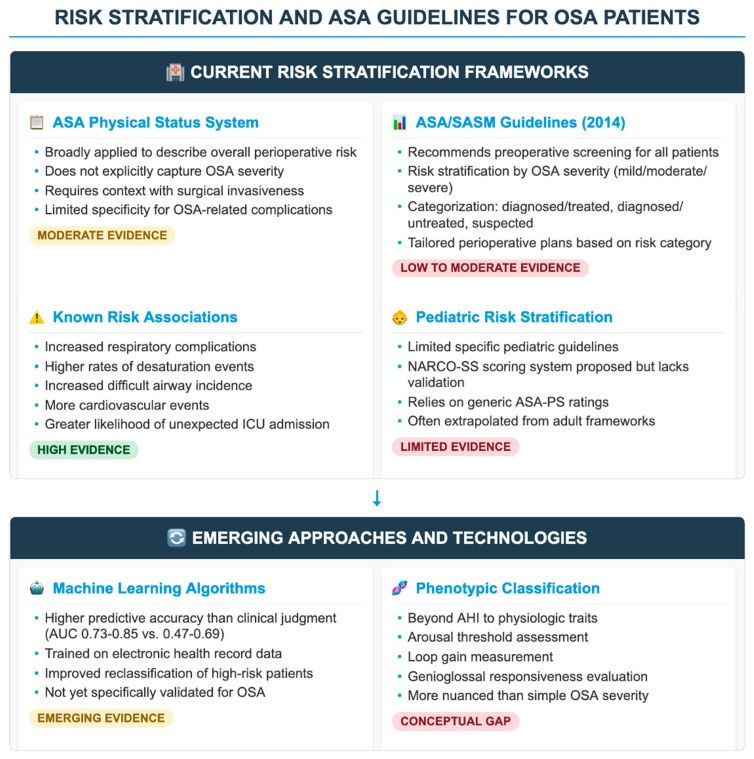
Risk stratification, emerging approaches, and level of evidence of literature for pediatric and adult OSA patients. The figure provides an insight into the current risk stratification strategies both in adult and pediatric OSA patients, as well as the emerging approaches including gaps in knowledge. ASA: American Society of Anesthesiology; SASM: Society of Anesthesia and Sleep Medicine; OSA: obstructive sleep apnea; ICU: intensive care unit; AUC: area under the curve; AHI: apnea–hypopnea index; NARCO-SS: neurological airway respiratory complication–sleep score.

Risk stratification in perioperative OSA management aims to identify patients at elevated risk of adverse events, yet substantial debate persists over whether existing frameworks—and, in particular, the ASA Physical Status system and related guidelines—offer sufficient granularity and clinical utility. The ASA Physical Status (PS) Classification is broadly applied to describe overall perioperative risk, including in patients with OSA. However, the ASA itself cautions that PS alone does not reliably predict perioperative complications unless contextualized with surgical invasiveness, patient frailty, and comorbidities [24]. This limitation becomes especially salient given that OSA severity is not explicitly captured by ASA-PS: two patients with identical ASA-PS status could have vastly different OSA risks (e.g., mild vs. severe AHI) [24]. ASA and SASM guidelines advocate for preoperative screening, risk stratification by OSA severity, and tailored perioperative care plans including avoidance of sedative premedication, use of regional anesthesia when appropriate, and postoperative monitoring or CPAP where indicated [7,24]. These are well-meaning recommendations but—critically—the evidence base underpinning many of these recommendations is predominantly low to moderate quality and derived mainly from observational studies or consensus rather than prospective randomized trials [22,25] (Figure 1).

Meta-analyses report that perioperative OSA is associated with increased respiratory complications, desaturation, difficult airway, cardiovascular events, and unexpected ICU admissions [7,8]. However, these reviews also highlight heterogeneity between studies, retrospective designs, and confounding by obesity and comorbidity burden, which limits causal inference and raises questions about the incremental value of OSA-specific stratification beyond established risk factors [7,8]. The task forces have attempted to distinguish categories such as “diagnosed and treated,” “diagnosed but untreated,” and “suspected OSA” to guide decisions—such as whether elective procedures should be delayed or enhanced monitoring deployed [25]. Yet, critics argue that such coarse categorizations may over-simplify diverse patient profiles and that recommendations such as delaying surgery for further PSG evaluation may lead to unnecessary delays or resource strain without clear outcome improvements unless severe hypoventilation or pulmonary hypertension is present [21,22]. Furthermore, ASA guidelines do not incorporate phenotypic or functional traits like arousal threshold, g loop gain, or genioglossal responsiveness that increasingly appear relevant for anesthetic risk—especially in stratifying risk of respiratory depression or difficult airway events [25]. This is a key conceptual gap: reliance on AHI as a severity metric may oversimplify the complex physiologic substrates underlying perioperative risk [22]. In pediatric practice, ASA recommendations are similarly limited. The guidelines reference pediatric patients but do not provide age-specific risk stratification parameters beyond generic ASA-PS ratings and procedural context [7]. Emerging pediatric scoring systems, such as the NARCO-SS (neurological airway respiratory complication–sleep score), have been proposed to stratify pediatric surgical risk more objectively based on craniofacial, obesity, and respiratory parameters, but lack broad validation and integration into formal perioperative protocols [16]. This highlights a broader issue; pediatric OSA risk stratification remains largely extrapolated from adult frameworks, ignoring physiologic and phenotypic differences. Table 2 provides a summary of the possible perioperative strategies in both pediatric and adult OSA patients according to the phenotype. Attention has also shifted to the integration of novel predictive models. Some centers have trialed machine learning algorithms trained on electronic health record data to estimate the risk of postoperative complications more accurately than clinical judgment alone [23,26]. For instance, pilot comparisons of algorithmic predictions versus clinician assessments showed higher AUC (~0.73–0.85) with algorithm outputs than with clinician estimates (AUC ~0.47–0.69), and model use was associated with improved reclassification of patients at risk of complications such as acute kidney injury and prolonged ICU admission [23]. Although these models were not specific to OSA, they underscore the potential for data-driven stratification that transcends ASA-PS or simple OSA severity scores. Critics also contend that stratification efforts lack integration with actionable perioperative pathways. Identifying a “high-risk” patient without a concrete protocol—e.g., clear thresholds for postoperative CPAP, ICU transfer, or sedation modifications—renders stratification less meaningful. Systematic reviews stress that while risk scores and guidelines recommend certain interventions (e.g., CPAP initiation, extended monitoring), only a minority of studies directly link these actions to improved morbidity or mortality outcomes [8,9]. This suggests a disconnection between risk identification and actual risk mitigation.

## 6. Anesthetic Induction and Airway Management

Anesthetic induction in patients with OSA remains one of the most hazardous phases of the perioperative journey. While expert opinion supports certain tactics, real-world data often expose gaps, physiological nuances, and controversies that challenge conventional wisdom (Table 3).

Patients with severe OSA face a 3- to 4-fold increased risk of difficult mask ventilation and intubation compared to non-OSA controls, based on observational and registry evidence [24]. Notably, a retrospective registry identified that first-pass success with conventional direct laryngoscopy was reduced by approximately 37% in patients with AHI > 30 [24]. These findings corroborate guidance from airway management task forces that advocate for advanced planning and readiness for alternate strategies. Traditional anatomical predictors—such as Mallampati score, thyromental distance, and neck circumference—often lack predictive power in this cohort. A recent review showed that STOP-Bang scores correlate poorly with actual intubation difficulty, underscoring that dynamic collapse risk is not adequately captured by static anatomical measures [7]. Videolaryngoscopy (VL) has emerged as a transformative tool in OSA airway management. A systematic review revealed that VL significantly reduces time to intubation, improves first-attempt success, and decreases ancillary maneuvers versus direct laryngoscopy—even in high-risk cohorts [25]. Another comprehensive review specifically emphasizes that VL consistently enhances glottic view while maintaining physiologic head–neck alignment, making it especially advantageous in OSA patients [25]. Another game-changing strategy is high-flow nasal oxygen (HFNO). A recent single-center randomized controlled trial showed HFNO at 60 L/min extended safe apnea time from approximately 4.2 to over 18 min, improved minimum SpO_2_ values, and lowered desaturation hazard (HR 0.071, 95% CI 0.021–0.222) compared with no supplemental oxygen [22]. A complementary meta-analysis involving obese or OSA-like patients similarly reported prolonged apnea duration—although not necessarily reduced hypoxemia—suggesting a physiological benefit tempered by heterogeneous outcomes [21]. Drug-induced sleep endoscopy (DISE), used to simulate natural airway collapse, also has direct anesthetic implications. A recent systematic review comparing DISE with natural sleep examinations affirmed that propofol- or midazolam-induced sedation reliably reproduces airway obstruction patterns—though dexmedetomidine may offer less respiratory depression with different hemodynamic profiles [27]. The procedure alters surgical planning in nearly half of pediatric cases, but also introduces airway risk unless conducted by teams with airway expertise [26].

In pediatric OSA with craniofacial anomalies, airway anatomy poses additional risk. Published series demonstrate that adolescents with OSA and severe anatomical features encountered difficult mask ventilation in ~18% and difficult intubation in ~13%, with awake fiberoptic techniques often preferred [23]. In pediatric patients, awake fiberoptic intubation poses unique challenges due to limited cooperation and higher sensitivity to sedatives. Recent randomized controlled trials comparing dexmedetomidine and remifentanil for sedation during airway procedures have shown that dexmedetomidine provides more stable oxygenation, with fewer apnea episodes and higher minimum SpO_2_ values, while maintaining adequate conditions for intubation [27]. These findings suggest that dexmedetomidine may be preferable in children at high risk of perioperative desaturation, although potential side effects such as bradycardia and delayed recovery require careful monitoring. Incorporating such evidence allows for more nuanced recommendations in pediatric OSA patients, moving beyond retrospective registries toward evidence-based sedation strategies. These data emphasize the need for phenotype-guided induction strategies customized to developmental and anatomic risk. Emerging airway adjuncts—including hyperangulated VL blades (GlideScope^®^, C-MAC^®^ D-blade^®^, McGrath^®^), channeled optical laryngoscopes (Airtraq^®^), and bougie/coude-tip devices—show promise. Multiple comparative and crossover trials report improved first-attempt success and better glottic visualization with hyperangulated devices in severe OSA airways compared to direct laryngoscopy [25]. However, head-to-head randomized trials specifically in the OSA population remain rare. The role of sedation depth and drug choice also presents real-world dilemmas. Retrospective analyses suggest that sedative combinations such as midazolam plus fentanyl more frequently precipitate hypoxic episodes in OSA patients—even when not statistically significant compared to controls—underscoring the need for conservative dosing and vigilant monitoring [23]. Techniques to mitigate physiologic vulnerability are gaining traction. Prolonged apneic oxygenation through HFNO provides clinicians with an additional buffer before desaturation, especially important in OSA patients with low functional residual capacity and high oxygen consumption [22]. Yet inconsistent adoption, varied protocols, and concerns about delayed airway intervention mean HFNO implementation is uneven. While awake fiberoptic intubation (AFI) continues to be the gold standard for high-risk OSA patients, successful execution hinges on technique. Techniques such as the “jaw-thrust first” maneuver and altered head extension, as validated by radiographic studies, significantly improve pharyngeal space and AFI success in OSA subjects [28]. Topical anesthesia using “spray-as-you-go” lidocaine is particularly effective, achieving high success rates (≈94%) with minimal sedative requirements [29]. Sedation agents also play a critical role; dexmedetomidine preserves respiratory drive and pharyngeal tone, and in a randomized comparison with remifentanil for awake AFI in severe OSA was associated with higher minimum saturations (94% vs. 88%) and fewer apnea episodes (7% vs. 40%) [30]. Conversely, remifentanil infusions at low doses allow precise sedation but carry a heightened risk of respiratory compromise and require continuous monitoring [31].

## 7. Opioids, Sedation, and Respiratory Depression

Postoperative analgesia in OSA patients poses a paradox: opioids effectively control pain but potentiate respiratory depression, especially in those with altered chemoreflex sensitivity or high arousal thresholds. Furthermore, intermittent hypoxia and sleep fragmentation—hallmarks of OSA—may amplify pain perception and opioid sensitivity simultaneously, creating a complex risk profile [27].

### 7.1. Adult Physiology and Opioid Sensitivity

Opioids suppress central ventilatory drive and impair upper airway patency by reducing genioglossus neuromuscular tone, thus exacerbating OSA severity [1]. Laboratory studies indicate that individuals with untreated OSA display greater ventilatory depression even with low opioid doses compared to non-OSA controls, likely due to blunted chemoreflex responsiveness [16]. Despite these clear physiologic vulnerabilities, randomized trials evaluating tailored opioid dosing in OSA cohorts are virtually absent; most evidence comes from observational studies or ICU models, limiting clinical applicability [7].

### 7.2. Opioid-Sparing Strategies in Adults

Recently, opioid-sparing anesthesia–analgesia (OSA-A) protocols have been increasingly applied in high-risk surgeries. A matched-cohort study on open thoracotomy patients revealed that OSA-A (without regional blocks) significantly reduced morphine consumption, pain scores in the first 48 h, PACU time, nausea/vomiting, and accelerated gastrointestinal recovery compared to opioid-based anesthesia (OBA-A) [2,6]. A parallel randomized trial in laparoscopic cholecystectomy confirmed that opioid-free anesthesia (OFA) was noninferior to OSA in pain control, with faster bowel recovery and less postoperative nausea, although early pain in PACU was modestly higher [13]. Meta-analyses support that multimodal techniques—including ketamine, lidocaine, magnesium, dexmedetomidine, NSAIDs, and acetaminophen—can decrease opioid use and its adverse effects without worsening pain [15,19,32].

### 7.3. Mechanistic Underpinnings and Phenotype-Specific Risk

The interaction between OSA trait phenotypes and opioid effects is increasingly elucidated. Patients characterized by low loop gain, high arousal threshold, and blunted chemosensitivity may tolerate pain better but are more vulnerable to opioid-induced ventilatory impairment [27]. Furthermore, intermittent hypoxia may paradoxically lower opioid analgesic thresholds while intensifying respiratory risk, producing a delicate analgesia-depression trade-off.

## 8. Intraoperative Ventilation and Monitoring

Intraoperative ventilation strategies and respiratory monitoring in OSA patients remain fraught with uncertainty. While the concept of lung-protective ventilation is well established in general anesthesia, its specific application and efficacy in OSA cohorts are underexplored, leaving substantial gaps in evidence and controversy.

### 8.1. Lung-Protective Ventilation (LPV)

LPV is defined by low tidal volumes (6–8 mL/kg predicted body weight) and moderate positive end-expiratory pressure (PEEP), and it has been associated with reduced risk of postoperative pulmonary complications (PPCs) in obese and surgical patients broadly [33]. However, obesity-related OSA patients present additional mechanical challenges such as atelectasis, decreased compliance, and increased airway collapse that may require adjusted PEEP levels. The PROBESE trial, involving obese surgical patients, randomized high PEEP (12 cmH_2_O) plus recruitment maneuvers versus low PEEP (4 cmH_2_O); however, results remain pending, and subgroup analyses involving OSA were not predefined [32]. The balance between alveolar recruitment and hemodynamic compromise remains debated, especially in patients with high BMI and OSA. Some studies suggest that moderate PEEP (8–10 cmH_2_O) during anesthesia may improve oxygenation and lung mechanics in obese patients with OSA, but excessive PEEP could impair venous return and lead to hypotension—particularly in intrathoracic surgery or in small pediatric patients with limited cardiovascular reserve [33,34]. Consensus on optimal PEEP settings in OSA is thus lacking, and standard LPV protocols may require phenotype-adjusted modifications.

### 8.2. Recruitment Maneuvers (RMs)

RMs could theoretically reverse atelectasis in OSA patients more effectively than in non-OSA individuals, but their role remains unsettled. Repetitive RMs may benefit patients with severe OSA and baseline low functional residual capacity; yet the potential for overdistension, surgical field interference, and cardiovascular perturbation limits their widespread adoption [34]. There is an urgent need for randomized controlled data specifically addressing RM frequencies, pressure levels, and patient profiles among OSA populations.

### 8.3. Intraoperative Monitoring

Standard intraoperative monitoring includes pulse oximetry and capnography; however, capnography—though often undervalued—is critically important in identifying early respiratory compromise. In sedated OSA or obese patients, waveform flattening and rising end-tidal CO_2_ often precede desaturation by two to three minutes, whereas pulse oximetry typically lags especially under supplemental oxygen [35]. A phase II trial comparing nasal pressure signals versus capnography during propofol–fentanyl sedation showed comparable predictive ability for desaturation, with nasal pressure detecting hypopnea or airway obstruction prior to SpO_2_ declines [36]. These findings suggest that multimodal respiratory monitoring—including nasal pressure, volumetric capnography, and pulse oximetry—could offer earlier warning and improved safety in OSA patients. Implementation studies in PACU settings—particularly nurse-initiated capnography protocols—demonstrated near-universal nurse compliance and rapid escalation for abnormal etCO_2_ excursions in OSA patients, leading to earlier interventions compared to reliance on pulse oximetry alone [37]. A systematic review of continuous capnography versus intermittent pulse checks confirmed significant reduction in adverse respiratory events and near-miss events, especially in patients at high OSA risk [35,37]. Despite these benefits, capnography remains inconsistently implemented, partly due to lack of standardized workflows and training in many centers.

Even though expert consensus supports LPV, PEEP optimization, and capnographic surveillance in high-risk OSA patients, few studies link these measures directly to improved clinical outcomes in this subgroup. Most data derive from observational cohorts or perioperative improvement projects—not randomized OSA-specific trials [33,34,35,36,37]. Standard ventilation protocols may fail to account for individual phenotypic differences, such as OSA severity, BMI, lung compliance, and arousal threshold. Children with OSA often have reduced FRC and elevated airway resistance, so LPV with low tidal volumes may inadvertently risk hypoventilation unless PEEP is carefully titrated. No pediatric OSA study has systematically evaluated intraoperative ventilation protocols: current practices are mostly extrapolated from adult data or institutional conventions. This demands a phenotype-based approach with prospective evaluation.

## 9. Postoperative Complications

Postoperative respiratory complications in OSA patients pose a profound risk—and accumulating evidence indicates that traditional PACU monitoring protocols alone may fall short. A systematic review of nearly 28,000 ambulatory surgery patients found OSA associated with a 1.65-fold increased risk of respiratory events and a 3.3-fold higher requirement for airway interventions—even in brief procedures under light sedation [38]. This underscores that even seemingly low-risk surgical contexts demand enhanced vigilance. A comprehensive meta-analysis of high-severity OSA cases revealed respiratory depression episodes in up to 17% of patients during the first 24 h postoperatively, often occurring after PACU discharge, coinciding with peak opioid effect [39]. These delayed events expose a vulnerability window that standard PACU surveillance may inadequately capture. Implementation of nurse-initiated capnography protocols within PACU settings has demonstrated remarkable feasibility and efficacy. In one project targeting STOP-Bang-positive patients, compliance approached 100%, and timely recognition of EtCO_2_ excursions triggered immediate escalations—though sample sizes were small [40]. Another randomized experience in postoperative orthopedic patients comparing continuous capnography with intermittent pulse oximetry revealed a 5.8-fold higher detection rate of respiratory depression events when monitoring EtCO_2_ [41,42]. Findings from the SASM OSA Death & Near-Miss Registry further reinforce the stakes as among 66 critical events 76% occurred within 24 h, 56% took place on regular wards, while another 21% occurred at home, with most events tied to unwitnessed periods, lack of supplemental oxygen, and absence of respiratory support [43]. Postoperative supplemental oxygen therapy may improve oxygen saturation and reduce apnea–hypopnea index without prolonging apneic episodes—but by masking hypercarbic respiratory depression, it may delay recognition absent capnographic surveillance [11]. In pediatric OSA patients, the risk persists; nighttime recurrent desaturation post-adenotonsillectomy continues into unmonitored hours, while most pediatric wards rely solely on intermittent SpO_2_ checks, leaving apnea events—and potential respiratory decline—undetected. Emerging remote monitoring technologies, such as nasal pressure sensors and PPG-derived respiratory rate monitoring, are showing promise for earlier detection of apnea or hypoventilation—often minutes before SpO_2_ drops—potentially extending surveillance beyond PACU into wards or home settings [5,44]. In the pediatric setting, post-adenotonsillectomy monitoring remains particularly controversial, with no universally accepted thresholds for escalation of care. While some guidelines extrapolate from adult criteria, prospective pediatric cohort studies suggest that children with severe OSA who experience recurrent desaturations below 85–90% in the early postoperative period are at highest risk of adverse events and may benefit from ICU-level monitoring. Recent evaluations of high-flow nasal cannula (HFNC) therapy in this population have shown reductions in desaturation episodes and unplanned ICU transfers, supporting its role as a feasible intermediate step between standard oxygen therapy and CPAP. These findings emphasize the need for pediatric-specific protocols that integrate both clinical risk factors and objective saturation thresholds to guide safe postoperative disposition.

## 10. Role of CPAP and Other Airway Adjuncts in the Perioperative Period

The perioperative use of continuous positive airway pressure (CPAP) and alternative airway adjuncts in OSA management remains a deeply debated and evolving domain. While CPAP is the cornerstone therapy for chronic OSA, its perioperative utility—timing, indications, compliance, and effectiveness—is shaped by inconsistent evidence and operational challenges. Several randomized studies have demonstrated that preoperative CPAP use improves oxygenation, reduces AHI, and augments postoperative respiratory stability. For instance, adult patients compliant with CPAP for at least 14 nights before elective surgery exhibited significantly fewer postoperative desaturation events and shorter PACU stays compared to non-users [45]. However, the benefit becomes ambiguous when CPAP is initiated just hours before surgery, raising questions about adequate preconditioning time. Postoperative-CPAP-only initiation has been trialed, with mixed results. A prospective cohort comparing patients started on CPAP immediately in PACU versus controls found decreased respiratory event rates and improved oxygen saturation—but without a robust reduction in ICU admission or length of stay [46]. Observational data further suggest that only patients with moderate-to-severe untreated OSA (AHI ≥ 30) derive substantial benefit: patients with milder disease saw no clear perioperative improvement [47,48]. Challenges in perioperative CPAP implementation include logistical and compliance issues. Many OSA patients are non-compliant with CPAP in their home setting, and bringing equipment into the hospital may lead to resource bottlenecks or non-adherence. A survey of surgical centers revealed that less than 50% had standard protocols for perioperative CPAP initiation; compliance rates wavered, particularly in pediatric or ambulatory contexts [49]. Beyond CPAP, high-flow nasal cannula (HFNC) and nasal bilevel positive airway pressure (BiPAP) have emerged as adjuncts. A recent randomized pilot in obese adult surgical patients indicated that prophylactic HFNC post-extubation reduced early desaturation episodes compared to standard oxygen, though data in OSA-specific cohorts are sparse [50]. BiPAP has shown greater efficacy in addressing postoperative hypercapnia and residual apnea compared with CPAP, especially in patients with comorbid obesity hypoventilation syndrome (OHS) and OSA [51]. In pediatric OSA, CPAP is rarely initiated perioperatively unless the child is already a home user. Instead, nasal cannula high-flow and supplemental oxygen are the default, despite limited outcome data. Retrospective evaluation of pediatric adenotonsillectomy patients demonstrated that children with severe preoperative OSA who received overnight HFNC had fewer desaturations and reduced ICU transfers—though inherent selection bias and lack of control group limit interpretability [52]. Phenotypic considerations are pivotal as patients with predominantly anatomical airway collapse (e.g., hypertrophic tonsils) may benefit more from CPAP or BiPAP perioperatively, while those with high loop gain or low arousal threshold may derive less advantage and may experience pressure-related discomfort or central apnea unmasking. This nuance is seldom integrated into guidelines or perioperative protocols. Controversies remain in the timing of CPAP initiation. Delaying surgery to allow a CPAP acclimatization period has been criticized as potentially unnecessary in asymptomatic patients with mild disease, creating logistical delays and potential anxiety without demonstrated outcome gain [45,47]. Conversely, immediate postoperative CPAP initiation in previously non-compliant individuals may provoke intolerance or airway dryness, leading to low adherence just when support is most needed. Implementing practical protocols requires clarity in threshold AHI, timing relative to surgery, CPAP settings, and patient education. A standardized protocol from a tertiary center mandated preoperative verification of CPAP prescription, encouraged at least one night of in-hospital usage before elective surgery, and provided PAP assistance postprocedure: their retrospective evaluation showed fewer airway interventions and unplanned inpatient stays in CPAP-compliant patients (AHI ≥ 15) compared to non-users (3.7% vs. 9.8%) [49]. However, lack of prospective validation limits generalization. Finally, future adjuncts include oral appliances, nasal EPAP (expiratory positive airway pressure), and positional supports. These tools remain largely experimental in the perioperative OSA setting but may offer alternatives when CPAP setup is not feasible. A pilot study in mild-to-moderate adult OSA patients showed that EPAP reduced AHI by ~50% overnight: its perioperative value is untested but potentially promising in low-severity scenarios or for short procedures [1].

## 11. Conclusions

The perioperative management of OSA patients—both adult and pediatric—remains a domain marked by clinical complexity and limited high-quality evidence. Despite the availability of screening tools and guidelines, significant gaps persist in their practical application, particularly regarding particular populations [53] and the integration of OSA physiological phenotypes into risk stratification, anesthetic planning, postoperative monitoring, and the association with hearing loss [19,20]. Throughout this review, it is clear that many perioperative strategies—such as CPAP application, opioid-sparing analgesia, and enhanced respiratory monitoring—offer plausible benefits, yet lack robust, phenotype-stratified validation. Pediatric care remains especially under-supported by prospective data. Variability in institutional resources, poor CPAP adherence, and inconsistent monitoring infrastructure further limit implementation of best practices.

### 11.1. Future Directions

Emerging technologies also represent a promising frontier for advancing perioperative care: smartphone-based tools for AHI estimation and artificial intelligence-driven monitoring systems using electronic health records or wearable sensors may allow real-time, individualized risk stratification and earlier detection of respiratory compromise. These innovations, once validated in prospective perioperative studies, could complement phenotype-based approaches and support decision-making in both adult and pediatric populations. Furthermore, the systemic consequences of OSA—including oxidative stress and inflammation—warrant closer integration into perioperative planning, particularly given their associations with neurocognitive decline and hearing loss. Looking forward, the field must evolve toward precise perioperative management. This demands prospective trials, phenotypic classification, and cross-disciplinary collaboration to align physiologic understanding with actionable protocols. Without such efforts, the current reliance on generalized algorithms and consensus statements will continue to limit safety and outcome optimization in this vulnerable patient population.

### 11.2. Limitations

This narrative review has some limitations that should be acknowledged. First, the analysis is constrained by the heterogeneity and predominantly retrospective nature of much of the available evidence, which limits the ability to draw causal inferences. Randomized controlled trials on anesthesiologic management of OSA, especially in pediatric populations, remain scarce, and many current recommendations are based on expert consensus rather than high-level evidence. Furthermore, most studies do not incorporate phenotypic variations in OSA, reducing the generalizability of findings across diverse patient subgroups. Finally, while this review aimed for comprehensive coverage, the exclusion of non-English literature and unpublished data may have limited the breadth of perspectives included.

## Figures and Tables

**Table 1:** Analytical comparison of OSA preoperative screening tools.

Tool/Score	Parameters Assessed	Sensitivity/Specificity	Clinical Feasibility	Limitations	Validated in Perioperative Context?
STOP-Bang	Snoring, tiredness, observed apnea, pressure (HTN), BMI, age, neck, and gender	Sensitivity: 84–95% (AHI ≥ 15); Specificity: ~43–56% [20]	High (self-report, quick)	Overestimates risk in obese patients; low specificity at cutoff ≥ 3	Yes—most widely studied
Berlin Questionnaire	Snoring severity, fatigue, HTN, and BMI	Sensitivity: ~68–86%; Specificity: ~39–59% [21]	Moderate (longer form)	Less practical in busy settings; moderate discriminatory power	Limited use in surgical patients
NoSAS Score	Neck circumference, BMI, age, sex, and snoring	Sensitivity: ~79%; Specificity: ~69% [22]	Moderate to high	Less validated outside sleep clinics; age-weighted risk may misclassify	Not widely adopted perioperatively
P-SAP Score (pediatric)	Snoring, apneas, growth delay, and tonsillar hypertrophy	Variable: sensitivity ~70–80% in small studies	Low (limited awareness)	Pediatric-specific but not standardized; not integrated in surgical workflows	No
Polysomnography (PSG)	Gold standard: AHI, desaturation, and arousals	Sensitivity/Specificity: 90–99% (diagnostic)	Low (costly, time-intensive)	Not feasible pre-op for most; delays surgery; poor access in many centers	Yes, but limited by access/time
Overnight oximetry	ODI and desaturation index	Sensitivity: 63–88%; Specificity: 40–70% [23]	Moderate to high (home-based)	Cannot detect hypopneas or arousals; may miss mild/moderate OSA	Growing interest in triage settings
Chung 2-step algorithm	STOP-Bang ≥ 3 → HCO_3_^−^ ≥ 28 or desaturation risk	Higher specificity than STOP-Bang alone [24]	Moderate (requires labs)	Requires labs; less suited to ambulatory or pediatric use	Partial validation

AHI = Apnea–hypopnea index; ODI = oxygen desaturation index. Sensitivity and specificity values may vary across studies and populations. Pediatric tools remain underdeveloped for perioperative application. There is no universal agreement on preoperative screening cutoffs for surgical triage.

**Table 2:** Phenotype-based perioperative strategies in adult and pediatric OSA patients.

OSA Phenotype	Clinical Features	Adult Perioperative Strategies	Pediatric Perioperative Strategies
High loop gain (ventilatory instability)	Hypersensitive ventilatory control and prone to periodic breathing	Avoid excessive supplemental O_2_ (may worsen instability); titrate opioids carefully; and enhanced capnography and overnight monitoring	Close observation in first 24 h; and avoid rapid discharge after adenotonsillectomy
Low arousal threshold	Arouse easily but prone to frequent desaturation under sedatives	Avoid benzodiazepines; consider dexmedetomidine or regional anesthesia; and cautious opioid dosing	Minimize sedative premedication; use short-acting anesthetics; and continuous pulse oximetry post-op
Poor muscle responsiveness (pharyngeal dilator dysfunction)	Increased risk of airway collapse and difficult mask ventilation	Prepare advanced airway plan (videolaryngoscopy, awake fiberoptic, HFNO); and avoid deep sedation without airway control	Anticipate difficult intubation in craniofacial anomalies; and consider inhalational induction to maintain spontaneous breathing
Anatomical collapsibility (e.g., obesity, tonsillar hypertrophy, retrognathia)	Structural upper airway narrowing	Positioning (ramped, jaw-thrust); PEEP during ventilation; and postoperative CPAP for moderate–severe OSA	Adenotonsillectomy is definitive therapy; perioperative HFNC or CPAP for severe cases; and ICU-level monitoring if desaturation persists
Blunted chemoreflex sensitivity	Reduced ventilatory response to hypoxia/hypercapnia and higher opioid sensitivity	Strict opioid-sparing/multimodal analgesia; and capnographic monitoring in PACU/ward	Careful opioid use; multimodal analgesia; and continuous monitoring overnight

OSA: Obstructive sleep apnea; HFNO: high-flow nasal oxygenation; HFNC: high-flow nasal cannula; PEEP: positive end-expiratory pressure; CPAP: continuous positive airway pressure; PACU: post-anesthesia care unit; ICU: intensive care unit.

**Table 3:** Airway management strategies for OSA patients.

Technique/Strategy	Clinical Application	Advantages	Limitations/Risks	Evidence Level/Consensus
Standard IV induction (propofol-based)	Routine adult induction; moderate OSA	Familiarity; rapid onset	Decreased pharyngeal tone; risk of apnea; worsens airway collapsibility [13]	Widely used; lacks OSA-specific RCTs
Inhalational induction (sevoflurane)	Pediatric patients; severe OSA with anticipated airway difficulty	Preserves spontaneous ventilation	Prolonged induction; risk of laryngospasm; limited adult data	Pediatric standard in select cases
Awake fiberoptic intubation (AFI)	Anticipated difficult airway; severe OSA	Maintains spontaneous breathing; avoids desaturation	Requires patient cooperation; time-consuming; requires expertise [27]	Strong expert consensus; limited trial evidence
Videolaryngoscopy (VL)	All OSA severities; standardizing in adult care	Improved glottic visualization; reduced cervical movement	Limited effectiveness in distorted anatomy; device cost; fogging or secretions [25]	High-level observational support; growing use
High-flow nasal oxygen (HFNO)	Preoxygenation and apneic oxygenation	Prolongs safe apnea time; improves desaturation threshold	May delay airway intervention; not suitable for full obstruction; variable flow tolerance [22]	Strong physiologic rationale; few RCTs in OSA
Use of neuromuscular blockers (NMBs)	Rapid-sequence induction; airway control	Reduces airway resistance; facilitates intubation	If not anticipated properly, can worsen airway obstruction in collapsed airway	Safe with expertise; controversial in OSA [29]
Jaw-thrust/positioning maneuvers	All inductions, especially in obese/OSA patients	Improves airway patency; simple and non-invasive	Transient effect; needs manual assistance; not definitive solution	Universal recommendation in OSA [27]
Avoidance of premedication (midazolam)	High-risk adult or pediatric patients with severe OSA	Reduces risk of sedation-related apnea	May increase anxiety or uncooperativeness pre-induction	Recommended by ASA/SASM for high-risk OSA [7]
Sedation with dexmedetomidine	Sedation during DISE or AFI; awake fiberoptic intubation	Preserves respiratory drive; less desaturation vs. opioids	Bradycardia; hypotension; delayed recovery [29]	Promising alternative; limited comparative studies

## Data Availability

No new data were created.

## Abbreviations

The following abbreviations are used in this manuscript:

AFI	Awake Fiberoptic Intubation
AHI	Apnea–Hypopnea Index
ASA	American Society of Anesthesiologists
AUC	Area Under the Curve
BiPAP	Bilevel Positive Airway Pressure
BMI	Body Mass Index
CHASE-OSA	(Pediatric OSA screening model—acronym not explicitly defined)
CI	Confidence Interval
CPAP	Continuous Positive Airway Pressure
DISE	Drug-Induced Sleep Endoscopy
EPAP	Expiratory Positive Airway Pressure
EtCO_2_	End-tidal Carbon Dioxide
HCO_3_^−^	Bicarbonate
HFNC	High-Flow Nasal Cannula
HFNO	High-Flow Nasal Oxygen
HR	Hazard Ratio
HTN	Hypertension
ICU	Intensive Care Unit
IV	Intravenous
LPV	Lung-Protective Ventilation
MAD	Mandibular Advancement Device
NARCO-SS	Neurological Airway Respiratory Complication–Sleep Score
NMB	Neuromuscular Blocker
NoSAS	(OSA screening score—acronym not explicitly defined)
NSAIDs	Non-Steroidal Anti-Inflammatory Drugs
OBA-A	Opioid-Based Anesthesia-Analgesia
ODI	Oxygen Desaturation Index
OFA	Opioid-Free Anesthesia
OHS	Obesity Hypoventilation Syndrome
OIRD	Opioid-Induced Respiratory Depression
OSA	Obstructive Sleep Apnea
OSA-A	Opioid-Sparing Anesthesia–Analgesia
PACU	Post-Anesthesia Care Unit
PAP	Positive Airway Pressure
PEEP	Positive End-Expiratory Pressure
PPCs	Postoperative Pulmonary Complications
PPG	Photoplethysmography
PS	Physical Status
PSG	Polysomnography
P-SAP	(Pediatric sleep apnea screening score—acronym not explicitly defined)
RCT	Randomized Controlled Trial
RM	Recruitment Maneuver
ROC	Receiver Operating Characteristic
SASM	Society of Anesthesia and Sleep Medicine
SDB	Sleep-Disordered Breathing
SpO_2_	Oxygen Saturation
STOP-Bang	Snoring, Tiredness, Observed apnea, Pressure (HTN), BMI, Age, Neck circumference, Gender
VL	Videolaryngoscopy
